# Discrepancies between *VEGF* −1154 G>A Polymorphism Analysis Performed in Peripheral Blood Samples and FFPE Tissue

**DOI:** 10.3390/ijms150813333

**Published:** 2014-07-30

**Authors:** Giorgia Marisi, Alessandro Passardi, Daniele Calistri, Wainer Zoli, Dino Amadori, Paola Ulivi

**Affiliations:** 1 Biosciences Laboratory, Istituto Scientifico Romagnolo per lo Studio e la Cura dei Tumori (IRST) IRCCS, 47014 Meldola (FC), Italy; E-Mails: giorgia.marisi@irst.emr.it (G.M.); daniele.calistri@irst.emr.it (D.C.); wainer.zoli@irst.emr.it (W.Z.); 2 Department of Medical Oncology, Istituto Scientifico Romagnolo per lo Studio e la Cura dei Tumori (IRST) IRCCS, 47014 Meldola (FC), Italy; E-Mails: alessando.passardi@irst.emr.it (A.P.); direzione.scientifica@irst.emr.it (D.A.)

**Keywords:** formalin fixed paraffin-embedded (FFPE) tissue, metastatic colorectal cancer (mCRC), peripheral blood, single nucleotide polymorphisms (SNPs), vascular endothelial growth factor (*VEGF*)

## Abstract

Single nucleotide polymorphisms (SNPs) may be associated with the response or toxicity to different types of treatment. Although SNP analysis is usually performed on DNA from peripheral blood, formalin fixed paraffin-embedded (FFPE) tissue is often used for retrospective studies. We analyzed *VEGF* (−2578C>A, −1498C>T, −1154G>A, −634C>G, +936C>T) and *eNOS* (+894G>T, −786T>C, VNTR (variable number of tandem repeats) 27bp intron 4) polymorphisms by direct sequencing or Real Time PCR in 237 patients with advanced colorectal cancer. Peripheral blood was used for 153 patients, whereas only FFPE tumor tissue was available for 84 patients. All SNP frequencies were in Hardy-Weinberg Equilibrium (HWE), with the exception of *VEGF* −1154, which was only in HWE in peripheral blood specimens. We therefore analyzed this SNP in DNA extracted from FFPE tumor tissue compared to FFPE healthy tissue and peripheral blood from 20 patients. Numerous heterozygous patients in peripheral blood DNA were homozygous for the A-allele in both tumor and healthy FFPE tissues. Our findings indicate that, although FFPE tissue might be a suitable specimen for genotyping, *VEGF* −1154 does not give reliable results on this type of material. As other SNPs may also have this limitation, genotype concordance should first be confirmed by comparing results obtained from FFPE and fresh sample analyses.

## 1. Introduction

In some pharmacogenetic studies, variations in DNA sequences have been associated with human disease development and response to drugs or other agents. In particular, some SNPs have been identified as useful predictive biomarkers of response to treatment in a number of pharmacogenetic studies [1,2], suggesting that the analysis of these polymorphisms could be useful to reduce the risk of adverse drug events and to improve the benefit from therapy. The majority of pharmacogenetic analyses have been carried out on germline DNA extracted from peripheral blood as it is easily obtained and generates large amounts of high quality DNA [3].

When peripheral blood is not available, especially in retrospective studies, archived formalin fixed paraffin-embedded (FFPE) tissue from either bioptic or resection specimens can also be used.

The quality of DNA isolated from FFPE tissue depends on the duration of formaldehyde fixation and on the formaldehyde buffer used [4]. Fixation can cause cross-linking and damage of DNA isolated from FFPE, creating problems with the amplification reaction and primer template recognition. Amplicons must thus be short and primers custom-designed as close as possible to the region of interest [5,6,7,8].

FFPE material can also create mutation artifacts, e.g., artificial C-T or G-A transitions [9], which should be taken into consideration when FFPE tissue is the source of material for genotyping analysis, especially when the genotype is essential to have information on drug response or drug metabolism. Several studies have performed genotyping analyses comparing FFPE and blood samples, some observing concordance between the two biological samples [10,11,12,13,14], and others reporting discordant results [15,16].

In the present study we analyzed five SNPs of *VEGF* and three of *eNOS*, both genes involved in angiogenesis and both potential biomarkers for predicting response to anti-angiogenic drugs. Our aim was to verify the possibility of correctly detecting these polymorphisms in DNA extracted from tumor and normal FFPE tissue specimens, in a series of patients with metastatic colorectal cancer. To this purpose we compared the results of the analysis of SNPs obtained from FFPE DNA with those obtained from peripheral blood as reference.

## 2. Results and Discussion

### 2.1. Results

The genotype distributions of all selected polymorphisms from 237 patients were in HWE, with the exception of *VEGF* −1154 G>A (HWpval 5.8 × 10^−5^). The HWE was thus calculated separately in peripheral blood and tumor FFPE samples. *VEGF* −1154 was only in HWE in peripheral blood samples (HWpval 0.1222 *vs.* 1.8 × 10^−5^). Different genotype frequencies were observed in FFPE tumor samples, with a higher frequency of AA homozygotes (26%) than that found in peripheral blood (14%) and a lower frequency of the heterozygote genotype (24% *vs.* 39%, respectively) (Table 1).

**Table 1:** Genotype frequencies of all SNPs (single nucleotide polymorphisms) in peripheral blood and FFPE (formalin fixed paraffin-embedded) tumor samples.

*VEGF* Polymorphisms	Peripheral Blood *N* = 153	FFPE Tumor Tissue *N* = 84
*VEGF −2578 C>A*		
C/C	39 (25.5)	24 (28.6)
C/A	74 (48.4)	46 (54.8)
A/A	40 (26.1)	14 (16.7)
*VEGF −1498 C>T*		
C/C	38 (24.8)	17 (20.2)
C/T	76 (49.7)	41 (48.8)
T/T	39 (25.5)	26 (31)
*VEGF −1154 G>A*		
G/G	72 (47)	42 (50) *
G/A	59 (38.6)	20 (23.8) *
A/A	22 (14.4)	22 (26.2) *
*VEGF −634 C>G*		
G/G	71 (46.4)	30 (35.7)
G/C	58 (37.9)	43 (51.2)
C/C	24 (15.7)	11 (13.1)
*VEGF +936 C>T*		
C/C	108 (70.6)	65 (77.4)
C/T	42 (27.4)	17 (20.2)
T/T	3 (2)	1 (1.2)
n.e.	-	1
*eNOS −786 T>C*		
T/T	59 (38.6)	21 (25)
T/C	77 (50.3)	40 (47.6)
C/C	17 (11.1)	22 (26.2)
n.e.	-	1
*eNOS +894 G>T*		
G/G	69 (45.1)	35 (41.7)
G/T	63 (41.2)	39 46.4)
T/T	21 (13.7)	10 (11.9)
*eNOS VNTR 4a/b*		
4b/b	111 (72.5)	51 (60.7)
4a/b	42 (27.5)	26 (30.9)
4a/a	0	3 (3.6)
n.e.	-	4

* not in HWE (Hardy-Weinberg Equilibrium); n.e., not evaluable; VNTR, variable number of tandem repeats.

We therefore selected 20 individuals for whom peripheral blood and healthy and tumor FFPE tissue samples were available to verify the concordance of polymorphism results.

There was 100% concordance between the results obtained in FFPE tumor tissue-derived DNA and those obtained in peripheral blood DNA for all but *VEGF −*1154 G>A polymorphism. Homozygosity for GG and AA in peripheral blood was confirmed in FFPE tissue, whereas the heterozygous genotype GA was not. In particular, of the 10 patients who were heterozygous for this SNP in peripheral blood DNA, 5 were homozygous for the A-allele (AA) and 4 showed virtually no evidence of the G allele in FFPE tumor tissue-derived DNA. Therefore, only one patient was clearly heterozygous when DNA from FFPE tumor tissue was considered (Table 2).

**Table 2:** Discordant *VEGF* −1154 polymorphism genotypes in DNA extracted from peripheral blood and tumor and healthy FFPE tissue.

Samples	*VEGF* −1154 Peripheral Blood	*VEGF* −1154 Tumor FFPE Tissue	*VEGF* −1154 Healthy FFPE Tissue
1	G/A	G/A *	G/A *
2	G/A	A/A	G/A *
3	G/A	G/A *	A/A
4	G/A	A/A	A/A
5	G/A	A/A	G/A *
6	G/A	A/A	A/A
7	G/A	G/A *	G/A *
8	G/A	A/A	n.a.
9	G/A	G/A *	n.a.

n.a., not available; * G allele peak was almost undetectable.

**Figure 1:**
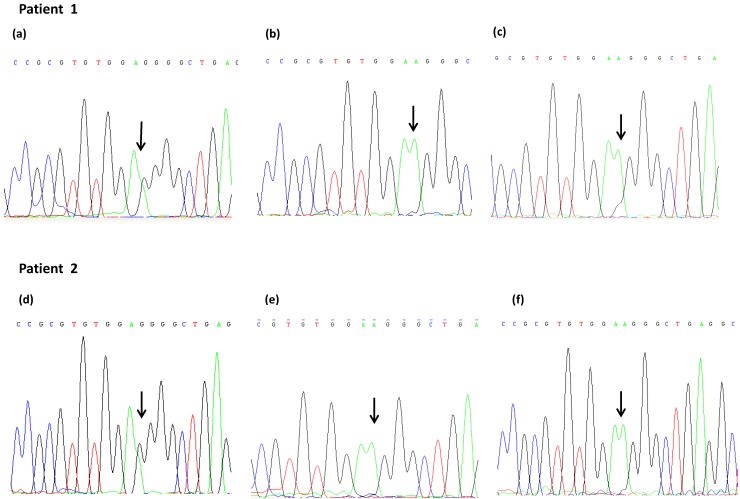
*VEGF*-1154 polymorphism analysis in two patients with discordant results, performed by direct sequencing on DNA extracted from peripheral blood (**a**,**d**) and from FFPE tumor (**b**,**e**) and healthy (**c**,**f**) tissue. The arrows indicate SNP localization.

We also analyzed DNA extracted from FFPE healthy tissue (reactive lymph node), processed in the same way as FFPE tumor tissue, to verify whether the loss of guanine was due to FFPE treatment or whether it was a characteristic of the tumor cells (Loss of Heterozygosity). FFPE healthy tissue was available in 7 of the 9 patients in whom discordant results were observed. This analysis of the DNA from healthy FFPE tissue revealed the same unclear results. Three samples were homozygous for the A-allele (AA) and 4 were weakly heterozygous GA (Table 2). An example of discordant results based on the analysis of the different biological samples in 2 patients is shown in Figure 1.

All the discordant results were confirmed in further independent experiments. The PCR reaction was repeated and the analysis was performed using the same technique.

### 2.2. Discussion

We demonstrated an absolute concordance between *VEGF* (−2578C>A, −1498C>T, −634C>G, +936C>T) and *eNOS* (+894G>T, −786T>C and VNTR 27bp intron 4) polymorphisms in DNA extracted from peripheral blood and in that from matched FFPE colorectal cancer tissue. Conversely, *VEGF* −1154 G>A results were not comparable, indicating that FFPE tissue is not suitable biological material on which to perform this specific polymorphism analysis.

In particular, the heterozygous genotype GA was difficult to interpret in FFPE tissue and in many cases the G allele was lost, with a consequent homozygous AA genotyping result. This was observed in both tumor and healthy FFPE tissue, suggesting that this alteration is due to a G-to-A transition, known to be induced by formalin fixation or paraffin treatment [9]. In a controlled study performed on frozen and formalin-fixed tissue, Williams and co-workers reported finding up to one mutation artifact per 500 bases in the latter material. Twenty-eight artificial mutations were detected, 27 of which occurred at the position of guanine or cytosine nucleotides and were C-to-T or G-to-A transitions. The remaining mutation was an A-to-T transversion [9].

A number of authors reported that *VEGF* −1154 G>A allele frequencies analyzed in blood samples were G/G in about 45%–50% of cases, G/A in 35%–40% and A/A in 10%–15% [17,18,19,20], in agreement with our results. Conversely, in FFPE tumor tissue, we observed higher frequencies of patients homozygous for the A-allele. Moreover, in contrast to our results, one study reported similar *VEGF* −1154 G>A allele frequencies in paraffin-embedded tumor tissue and in peripheral blood mononuclear cells of healthy controls [21]. The explanation for this discrepancy could lie in the different fixation procedures used, different pH values of fixatives, or specimen aging, all known to be involved in the induction of DNA alterations [22].

A number of works have also analyzed the concordance between other polymorphisms detected in DNA extracted from peripheral blood and FFPE tissue. Some described an absolute concordance between the two biological samples [10,11,12,13,14]. Before starting their studies, Rae and co-workers [10] and Wakatsuki and co-workers [13] examined a small sample set for each polymorphism to be analyzed, confirming a 100% genotype concordance rate between FFPE and whole blood samples. Conversely, discordant results were reported by other authors [15,16]; in particular, in van Huis-Tanja and co-worker’s prospective randomized study of 149 patients with advanced colorectal cancer characterized for several polymorphisms, 16 heterozygous patients appeared homozygous in FFPE colorectal tumor samples, and the most frequently observed genetic variations were C-to-T or G-to-A transitions (e.g., *ERCC2* G956A, *GSTP1* G342A and *ABCB1* C1236T). In particular, there was significant discordance for *GSTP1* (*glutathione S-transferase P*) between FFPE tissue and blood samples, the authors reporting a transition of the heterozygous to the homozygous wild-type genotype in 4 (6%) out of the 69 tumors of heterozygous patients [15].

Discrepancies between blood and tumor may be due to the loss of heterozygosity (LOH) in tumors [23]. If a tumor shows a loss of one of the two alleles (LOH), SNP analysis may result in misclassification of the patient’s drug metabolism phenotype [24]. Xie *et al.* [11] studied five SNPs, known to have LOH in breast cancer, in blood and normal FFPE tissue adjacent to breast tumors. 100% concordance was observed between the two specimens, indicating that adjacent normal tissue can provide accurate information on genotyping. Similarly to our study, Rae and co-workers [14] assessed the concordance between *CYP2D6* genotypes using 3 tissue sources (formalin-fixed, paraffin-embedded tumors [FFPETs]; formalin-fixed, paraffin-embedded unaffected lymph nodes [FFPELNs]; and whole blood cells) from 122 breast cancer patients, reporting that *CYP2D6* genotypes obtained from FFPETs and FFPELNs were more than 94% concordant with blood genotypes. In observing similar rates of discordance between FFPETs/FFPELNs and whole blood cells, they also refuted the hypothesis that breast tumor LOH causes misclassification when FFPETs are used. Similarly, our discordant findings on blood and FFPE samples cannot be attributed to tumor LOH as we obtained similar results from FFPE tumor and normal tissue analysis, suggesting that *VEGF* −1154 may be susceptible to damage induced by paraffin embedding.

Baak-Pablo and co-workers demonstrated that specific improvements in DNA amplification processes could optimize genotyping analysis from FFPE tissue [16]. In particular, a pre-amplification step using a SNP-specific Taqman assay proved capable of optimizing genotyping results. The authors compared the results from the analysis of both types of DNA (FFPE and blood samples) with or without pre-amplification in 22 patients. Four mismatches were found in three SNPs (*ABCB1* rs1128503 A>G, *p53* rs1042522 G>C, *ERCC2* rs1799793 C>T) when whole blood DNA was compared with non preamplified genotypes, and 100% concordance was observed between blood DNA (preamplified or nonpreamplified) and preamplified FFPE tissue-derived DNA [16]. The most frequently observed genetic variations were C-to-T and G-to-A transitions. These results suggest that SNP analysis optimization using a variety of techniques such as Real-time PCR, high throughput sequencing or SNP array, improved DNA amplification strategies, e.g., allele-specific preamplification, or a more accurate selection of primers and different methods of DNA extraction from paraffin-embedded tissues [5,25] could potentially reduce genotyping errors. However, paraffin-embedding induces permanent modifications and thus improved analysis techniques may not always overcome the problem. It is therefore necessary, before starting a study, to verify genotype concordance by evaluating each polymorphism to be analyzed in a small subset of FFPE and fresh samples. Analysis of the deviation of the genotype distribution from Hardy-Weinberg equilibrium should also be performed to guarantee the quality of primary genetic data [26].

## 3. Experimental Section

### 3.1. Samples and DNA Isolation

A total of 237 patients enrolled onto the phase III prospective multicenter randomized “Italian Trial in Advanced Colorectal Cancer (ITACa)” trial [27] were considered in this study after giving written informed consent for their biological material to be used for research purposes. The case series comprised 144 males and 93 females with a median age of 67 years (range 34–83). Peripheral blood samples were analyzed for 153 patients, whereas only paraffin-embedded tumor samples were available for 84 patients. A comparative analysis of 20 patients (13 males, 7 females, median age 68 years (range 48–81)) was carried out using peripheral blood, FFPE healthy tissue and FFPE tumor tissue.

Genomic DNA was extracted from peripheral blood samples by QIAamp DNA Mini Kit (Qiagen, Hilden, Germany) according to the Blood and Body Fluid Spin protocol. Genomic DNA was also extracted from tumor or non-tumor FFPE tissue. Five-μm tissue sections were collected in specific tubes for DNA extraction. Tumor cells were lysed in 50 mM of KCl, 10 mM of Tris-HCl pH 8.0, 2.5 mM of MgCl_2_ and Tween-20 (0.45%) in the presence of 1.25 mg/mL of proteinase K, overnight at 56 °C. Proteinase K was inactivated at 95 °C for 10 min and samples were then centrifuged twice at 6000 rpm to eliminate debris. DNA was purified using QIAamp DNA Micro kit (Qiagen) following the Cleanup of genomic DNA protocol. DNA quantity and quality were assessed by Nanodrop 1000 (Celbio, Milan, Italy).

### 3.2. Genotyping

Five *VEGF* polymorphisms (*VEGF* −2578C>A, −1498C>T, −1154G>A, −634C>G and +936C>T) and two *eNOS* polymorphisms (*eNOS* +894G>T and VNTR 27bp intron 4) were detected by standard PCR reaction and direct sequencing analysis using *3130 Avant Genetic Analyzer (*Applied Biosystems, Foster City, CA, USA). The *eNOS* −786T>C polymorphism was detected by Real-Time PCR (Taqman SNP Genotyping Assays, Assay ID C_15903863_10, Applied Biosystems). Cycling conditions were as follows: one minute at 95 °C for DNA denaturation, one minute at the annealing temperature specific for each pair of primers and one minute at 72 °C for the extension of PCR products, for 38 cycles. Some modifications of the PCR programs were required for the amplification of DNA from FFPE tissue, and inner primers for nested PCR were used for two SNPs (*VEGF* −1498 and *VEGF* −1154) (Table 3). After sequencing and genotyping, the Hardy-Weinberg equilibrium was determined using the Haploview program (Version 4.2) (Broad Institute of MIT and Harvard, Cambridge, MA, USA).

**Table 3:** Primer sequences for *VEGF* and *eNOS* SNPs.

SNPs	Location	RS-Number	Primers	Annealing Temperature	Product Size
*VEGF* −2578C>A	Promoter	rs699947	F: 5'-AAC-CTA-GCA-CCT-CCA-CCA-AA-3'	60 °C	268 bp
			R: 5'-GCT-GGT-TTC-TGA-CCT-GGC-TA-3'		
*VEGF* −1498C>T	Promoter	rs833061	F: 5'-AAG-CCC-ATT-CCC-TCT-TTA-GC-3'	60 °C	303 bp
			R: 5'-CTG-AGA-GCC-GTT-CCC-TCT-TT-3'		
*VEGF* −1498C>T nested *			F: 5'-ACA-GGG-AAG-CTG-GGT-GAA-T-3'	58 °C	235 bp
			R: 5'-CTG-AGA-GCC-GTT-CCC-TCT-TT-3'		
*VEGF* −1154G>A	Promoter	rs1570360	F: 5'-TTT-TCA-GGC-TGT-GAA-CCT-TG-3'	62 °C	264 bp
			R: 5'-ACG-ACC-TCC-GAG-CTA-CCC-3'		
*VEGF* −1154G>A nested *			F: 5'-TTT-TCA-GGC-TGT-GAA-CCT-TG-3'	60 °C	227 bp
			R: 5'-GAT-CCT-CCC-CGC-TAC-CAG-3'		
*VEGF* −634C>G	5'UTR	rs2010963	F: 5'-GGA-TTT-TGG-AAA-CCA-GCA-GA-3'	62 °C	224 bp
			R: 5'-CTG-TCT-GTC-TGT-CCG-TCA-GC-3'		
*VEGF* +936C>T	3'UTR	rs3025039	F: 5'-ACA-CCA-TCA-CCA-TCG-ACA-GA-3'	58 °C	226 bp
			R: 5'-CAG-GAA-TCC-CAG-AAA-TAA-AAC-TC-3'		
*eNOS* +894G>T	Exon 7	rs1799983	F: 5'-AAG-GCA-GGA-GAC-AGT-GGA-TG-3'	64 °C	319 bp
			R: 5'-GTT-GGG-GTG-TGG-GAT-CAG-3'		
*eNOS* VNTR 27bp	Intron 4	-	F: 5'-AAA-CTG-TGG-GGG-AGA-TCC-TT-3'	62 °C	544 bp
			R: 5'-GGG-CAG-CTT-GCT-TCT-CTT-AG-3'		

F, forward primer; R, reverse primer; UTR, untranslated region; * only used for DNA extracted from FFPE tissues.

## 4. Conclusions

Our results indicate that *VEGF* −1154 G>A analysis is not reliable in FFPE tissues and that homozygous AA and heterozygous GA genotypes may be over- and under-estimated, respectively. However, all other *VEGF* and *eNOS* polymorphisms were comparable in peripheral blood and FFPE samples, suggesting that FFPE tissue is a valuable source of biological material on which the majority of molecular studies can be performed. FFPE tissue could thus represent an alternative source of DNA for pharmacogenetic analysis in studies for which peripheral blood samples are not available, e.g., retrospective studies. However, it is nonetheless advisable to perform a matched analysis on FFPE and fresh/frozen samples for each polymorphism to be analyzed, especially those containing C or G alleles which may be modified by the FFPE procedure. Optimization of the methods used for DNA extraction and amplification could potentially overcome this problem.
